# Optimization of Single-Sensor Two-State Hot-Wire Anemometer Transmission Bandwidth

**DOI:** 10.3390/s8106747

**Published:** 2008-10-28

**Authors:** Paweł Ligęza

**Affiliations:** Strata Mechanics Research Institute Polish Academy of Sciences / Reymonta 27, 30-59 Kraków, Poland; E-mail: ligeza@img-pan.krakow.pl; Tel. +48-12-637-62-00; Fax: +48-12-637-28-84

**Keywords:** Hot–wire anemometry, temperature correction, frequency bandwidth, computer simulation, optimization

## Abstract

Hot-wire anemometric measurements of non-isothermal flows require the use of thermal compensation or correction circuitry. One possible solution is a two-state hot-wire anemometer that uses the cyclically changing heating level of a single sensor. The area in which flow velocity and fluid temperature can be measured is limited by the dimensions of the sensor's active element. The system is designed to measure flows characterized by high velocity and temperature gradients, although its transmission bandwidth is very limited. In this study, we propose a method to optimize the two-state hot-wire anemometer transmission bandwidth. The method is based on the use of a specialized constant-temperature system together with variable dynamic parameters. It is also based on a suitable measurement cycle paradigm. Analysis of the method was undertaken using model testing. Our results reveal a possible significant broadening of the two-state hot-wire anemometer's transmission bandwidth.

## 1. Introduction

Hot-wire anemometry is a method for indirectly measuring fluid flow velocity, based on monitoring thermal losses in a heated measuring element. The result is a function of not only the flow velocity, but also of certain other flow parameters. The measurement result is significantly affected by the fluid temperature [1-4]. Accordingly, hot-wire anemometric measurements of non-isothermal flows require the use of a temperature compensation or correction system. The review of the temperature compensation or correction methods presented in this study concerns hot-wire anemometers operating in the most commonly used constant-temperature mode.

The basic compensation system comprises a constant-temperature bridge circuit [5, 6]. A velocity sensor is in one branch, while a temperature compensation sensor is placed in the other branch of the bridge. The use of a compensation sensor with a resistance considerably higher than that of the velocity sensor is required. A modified compensation system for a constant-temperature hot-wire anemometer that is based on a bridge circuit [7] allows for the use of a temperature compensation sensor of any resistance. Another approach for temperature compensation is based on incorporating into the bridge circuit an electronic element the resistance of which is controlled by a signal from the separate temperature measurement circuitry [8]. For precise anemometric measurements, we developed an original non-bridge constant-temperature circuit with four-point measurement of the sensor resistance [9, 10]. Adding a temperature compensation sensor with an appropriate circuit allows for temperature compensation [11].

Temperature-correction of the output signal is based on the conversion of the signal generated by the constant-temperature system (without compensation) into the temperature-compensated signal [12]. In the basic system used for correcting the constant-temperature hot-wire anemometer output signal [13] the signal is corrected by a voltage that is proportional to the fluid temperature. Another solution to the temperature correction problem is a system that incorporates two hot-wire anemometers [14-16]. Importantly, both sensors operate in constant-temperature circuits, thereby allowing for the transmission of a wide range of frequencies for both velocity and temperature measurements. An interesting modification of the two-anemometer system is the two-state hot-wire anemometer, which operates on the basis of a periodically changing heating level from a single measurement sensor [17-19]. The flow velocity and fluid temperature are determined based on the steady-state output signals that correspond to two predefined levels of heating. The hot-wire anemometer must have the capability of changing the hot-wire anemometric sensor heating level. The great advantage of this system is the fact that the measurement ranges for temperature and velocity are restricted to the dimensions of a single active element of the hot-wire anemometric sensor. The system is perfectly suitable for measuring flows that are characterized by high velocities or temperature gradients. Typical applications include analysis of the velocity and temperature distribution in heat exchangers, analysis of flows around strongly heated elements, and analysis of heating and cooling systems. Nevertheless, the system is characterized by a very limited band of transmitted frequencies. Since the two-state hotwire anemometer offers a unique measurement instrument which features a wide range of applications, the optimization of its transmission bandwidth is of great interest.

The transmission bandwidth of the two-state hot-wire anemometer is dependent on the time needed for the signal to settle following the switch to a given sensor heating level. This time determines the maximum allowable frequency of switching between the sensor heating levels and thus also the frequency of flow parameter calculations. The solutions proposed to date are based on a classical constant-temperature system with fixed dynamic parameters. In such a system, the time required for establishing a steady state following changes in the sensor heating level is extremely dependent on both flow velocity and the actual heating level. In practice, optimizing the switching process via system regulation is possible only for a single velocity value. For other velocity values, the times needed for a signal to settle are suboptimal. As a consequence, to date we have used a low frequency of switching between heating levels, ranging from several Hz to several hundreds of Hz. This frequency was chosen so as to achieve steady state across a range of velocities.

In this study, we propose optimizing the two-state hot-wire anemometer transmission bandwidth by means of two approaches. Our optimization strategy relies on a specialized constant-temperature circuit with variable dynamic parameters and an appropriate measurement cycling strategy. In our solution, the constant-temperature circuit can be dynamically regulated. Immediately before switching over to a different heating level, the dynamic parameters of the constant-temperature circuit are stored in a way that allows us to calculate minimum time required to reach steady state. This regulation is performed based on flow velocity and heating level. The measurement cycle strategy is as follows:

▪ switch over to the preset heating level, and then determine flow velocity and temperature of the fluid based on the value of the sensor current recorded during the two previous steady state scenarios,
▪ a sequence of sensor current measurements is performed until the system reaches a steady state consistent with the stated criteria,
▪ given the previously calculated flow velocity and heating level, the optimal dynamic parameters of the constant-temperature circuit are calculated,
▪ the next sensor heating level is targeted and the cycle repeats.

The proposed circuit structure and measurement cycling strategy allow us to achieve a maximum switching frequency between the heating levels. Besides optimizing transmission bandwidth, we also minimize measurement errors. We created a computer simulation to test our proposed solution to optimize two-state hot-wire anemometer transmission bandwidth. We simulated system operation across a wide range of parameters and different operational modes.

### 2. Measurement methods for the two-state hot-wire anemometer

The two-state hot-wire anemometer allows for non-isothermal flow velocity and temperature measurements using a single measuring sensor. This sensor operates alongside the controlled constant-temperature hot-wire anemometric system [9]. The temperature of the sensor's active element periodically switches between two levels. This temperature must be significantly higher than the maximal fluid temperature. Following a switching routine, the sensor's steady-state current needs to be measured. The flow velocity and fluid temperature are determined based on results from measuring the current and previous measurement cycles. This method considerably reduces the effect of fluid temperature on the result of the velocity measurement. This study proposes the following measurement algorithm for the two-state hot-wire anemometer. Let us assume linear dependence between the sensor's active element resistance and its temperature:

(1) $$R=R_{0}\left(1+\alpha_{0}\left(T-T_{0}\right)\right),$$

where: R – sensor's resistance at the temperature T, R_0_ – sensor's resistance at the reference temperature T_0_, α_0_ – temperature coefficient of resistance at the reference temperature for the material of which the sensor is made. In order to describe the method, the model of the two-state hot-wire anemometer sensor may be assumed to be of the following form [20]:

(2) $$I^{2}R=I_{L}^{2}\left(R-R_{G}\right)\left[1+{\left(\frac{V}{V_{L}}\right)}^{n}\right]+I_{L}^{2}\tau_{L}\frac{\text{d}R}{\text{d}t},$$

where: I – sensor current, R_G_ – the sensor's resistance at the fluid temperature T_G_, V – the flow velocity of the fluid, I_L_, V_L_, τ_L_, n – model parameters, t – time. The coefficients of sensor heating relative to the reference temperature are as follows:

(3) $$\eta =\frac{R}{R_{0}},$$

(4) $$\eta_{G}=\frac{R_{G}}{R_{0}}$$

Now we assume that the flow velocity and fluid temperature between successive measurement cycles switch-over's do not change significantly. Additionally, we assume that the exponent *n* in the expression describing the sensor model is constant. Given these assumptions, in both phases of the measurement cycle the steady state of the sensor can be described by the following system of equations:

(5) $$I_{i}^{2}\eta_{i}={I_{Li}}^{2}\left(\eta_{i}-\eta_{G}\right)\left[1+{\left(\frac{V}{V_{Li}}\right)}^{n}\right],i=1,2.$$

Based on (5), the measured flow velocity *v* and the heating coefficient *η_G_* can be determined. Subsequently, the measured fluid temperature may be expressed by the relationship:

(6) $$\theta_{G}=T_{0}+\frac{\eta_{G}-1}{\alpha_{0}}$$

The general solution of the system of equations (5) is of the form:

(7) $$v={\left(\frac{1}{2\left(\eta_{2}-\eta_{1}\right)}\left(\frac{I_{2}^{2}\eta_{2}{V_{L2}}^{n}}{{I_{L2}}^{2}}-\frac{I_{1}^{2}\eta_{1}{V_{L1}}^{n}}{{I_{L1}}^{2}}-\left(\eta_{2}-\eta_{1}\right)\left({V_{L2}}^{n}+{V_{L1}}^{n}\right)++\sqrt{{\left(\frac{I_{2}^{2}\eta_{2}{V_{L2}}^{n}}{{I_{L2}}^{2}}-\frac{I_{1}^{2}\eta_{1}{V_{L1}}^{n}}{{I_{L1}}^{2}}\right)}^{2}-2\left(\eta_{2}-\eta_{1}\right)\left({V_{L2}}^{n}-{V_{L1}}^{n}\right)\left(\frac{I_{2}^{2}\eta_{2}{V_{L2}}^{n}}{{I_{L2}}^{2}}+\frac{I_{1}^{2}\eta_{1}{V_{L1}}^{n}}{{I_{L1}}^{2}}\right)+{\left(\eta_{2}-\eta_{1}\right)}^{2}{\left({V_{L2}}^{n}-{V_{L1}}^{n}\right)}^{2}}\right)\right)}^{\frac{1}{n}}$$

(8) $$\eta_{G}=\frac{1}{2\left({V_{L2}}^{n}-{V_{L1}}^{n}\right)}\left(-\frac{I_{2}^{2}\eta_{2}{V_{L2}}^{n}}{{I_{L2}}^{2}}+\frac{I_{1}^{2}\eta_{1}{V_{L1}}^{n}}{{I_{L1}}^{2}}+\left(\eta_{1}+\eta_{2}\right)\left({V_{L2}}^{n}-{V_{L1}}^{n}\right)++\sqrt{{\left(\frac{I_{2}^{2}\eta_{2}{V_{L2}}^{n}}{{I_{L2}}^{2}}-\frac{I_{1}^{2}\eta_{1}{V_{L1}}^{n}}{{I_{L1}}^{2}}\right)}^{2}-2\left({V_{L2}}^{n}-{V_{L1}}^{n}\right)\left(\eta_{2}-\eta_{1}\right)\left(\frac{I_{2}^{2}\eta_{2}{V_{L2}}^{n}}{{I_{L2}}^{2}}+\frac{I_{1}^{2}\eta_{1}{V_{L1}}^{n}}{{I_{L1}}^{2}}\right)+{\left({V_{L2}}^{n}-{V_{L1}}^{n}\right)}^{2}{\left(\eta_{2}-\eta_{1}\right)}^{2}}\right)$$

where the existence of a real solution is conditional on the denominators being non-zero and square roots being non-negative.

However, assuming that the sensor model parameters are independent of the heating level:

(9) $$I_{Li}=I_{L},\;V_{Li}=V_{L},\;i=1,2,$$

the solution takes the simplified form:

(10) $$v={\left(\frac{I_{2}^{2}\eta_{2}-I_{1}^{2}\eta_{1}}{I_{L}^{2}\left(\eta_{2}-\eta_{1}\right)}-1\right)}^{\frac{1}{n}}V_{L},$$

(11) $$\eta_{G}=\eta_{1}\eta_{2}\frac{I_{2}^{2}-I_{1}^{2}}{I_{2}^{2}\eta_{2}-I_{1}^{2}\eta_{1}}$$

The general equations (6), (7), (8) or the simplified expressions (6), (10), (11) allow us to determine the flow velocity and fluid temperature using the two-state hot-wire anemometer. Using the general equations, a higher precision measurement can be calculated; however, the required calculations in this case are much more time-consuming.

## 3. Dynamic model of the measurement system

**Figure 1.**
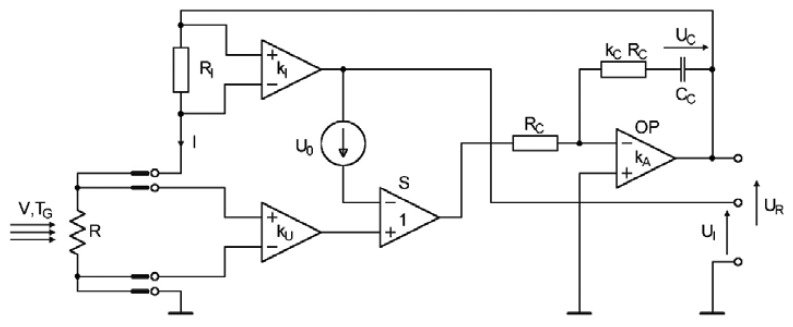
A simplified schematic diagram of the measuring system.

In order to simulate and optimize the model, we developed a dynamic mathematical model of the measurement system. The author's original non-bridge constant-temperature circuit that used a four-point measurement of sensor resistance was implemented in the two-state hot-wire anemometer [9]. This system enables us to eliminate the effect of the sensor feeding resistance on the heating coefficient. The implementation of this system results from our need to precisely set and maintain the measuring sensor heating coefficient. This is important to minimize measuring errors. However, one can also implement the classical constant-temperature bridge circuit. The simplified schematic diagram is shown in Figure 1.

The principle of operation is as follows: the voltage at the active element of sensor R is amplified by the differential amplifier k_U_. The voltage, proportional to the sensor current, on the R_I_ resistor is amplified by the differential amplifier k_I_. The output voltages of these amplifiers are compared by a unity gain differential amplifier S. The error voltage on the output of amplifier S is used to regulate the PI controller that includes operational amplifier OP. The controller supplies sensor R with a voltage U_R_ such that the error voltage tends toward zero. This allows for the maintenance of a constant sensor resistance. The voltage U_I_, which is proportional to sensor current, is the output signal of the system. The source of the offset voltage U_0_ enables the system to start up immediately it is switched on and it controls both the static and the dynamic parameters of the circuit.

Since the parameters of both the flow and the system may change across a wide range during testing, the use of a nonlinear model of the measuring system in the time domain has been suggested. The mathematical model of a measuring system as shown in Figure 1 is governed by equations that describes the system's components. Designations are made consistent with Figure 1. The following assumptions were made during model development:

▪ sensor resistance is a linear function of temperature consistent with Equation (1),
▪ the hot-wire anemometric sensor is described by the first-order model (2),
▪ the parasitic impedances are not taken into account,
▪ the operational amplifier of the controller is described by a first-order inertial model that takes into account the input resistance R_A_, the gain k_A_ and the time constant τ_A_,
▪ the differential amplifiers are characterized by a high input resistance and a non-inertial function,
▪ the gains k_U_ and k_C_ may be controlled using an external controlling signal,
▪ in the system, the signal variability is not constrained and the non-linear effects associated with the saturation of various electronic elements are not taken into account.

The time constant of the proportional-integrating controller is determined by the equation:

(12) $$\tau_{C}=k_{C}R_{C}C_{C}$$

Considering the aforementioned relationships and assuming that the state variables describing the system include voltages U_R_ and U_C_ and the sensor resistance R, analysis of the circuit in Figure 1 reveals a system of equations that describe the constant-bandwidth anemometer, written in the following form:

(13) $$\frac{dU_{C}}{dt}=\frac{-\left(R_{C}/R_{A}+1\right)U_{C}+\left(R_{C}/R_{A}+1-\left(k_{U}R-K_{I}R_{I}\right)/\left(R+R_{I}\right)\right)U_{R}+U_{0}}{\tau_{C}\left(R_{C}/R_{A}+1+1/k_{C}\right)},$$

(14) $$\frac{dU_{R}}{dt}=\frac{\left(1/k_{C}\right)U_{C}-\left(\left(1/k_{A}+1\right)/k_{C}+R_{C}/\left(k_{A}R_{A}\right)+1/k_{A}-\left(k_{U}R-k_{I}R_{I}\right)/\left(R+R_{I}\right)\right)U_{R}+U_{0}}{\tau_{A}\left[R_{C}/\left(k_{A}R_{A}\right)+1/k_{A}+1/\left(k_{A}k_{C}\right)\right]},$$

(15) $$\frac{\text{d}R}{\text{d}t}=\frac{U_{R}^{2}R/\left[I_{L}^{2}{\left(R+R_{I}\right)}^{2}\right]-\left(R-R_{G}\right)\left[1+{\left(V/V_{L}\right)}^{n}\right]}{\tau_{L}}$$

Notably, assuming a high value of the gain k_A_ and assuming that the voltage U_0_ tends to zero, in the steady state, equations (13) and (14) result in the approximation:

(16) $$R=R_{I}\frac{k_{I}}{k_{U}}$$

Therefore the steady state resistance of the sensor is a function of the k_I_-to-k_U_ ratio. In the system analyzed here, the heating level can be altered by adjusting the gain k_U_. On the other hand, the dynamic parameters of the system can be determined by adjusting the controller gain k_C_ [21, 22].

The output equation for the system model can be written in the form of the equation describing the relationship between sensor current and state variables:

(17) $$I=\frac{U_{R}}{R+R_{I}}$$

Equations (13), (14), (15) and (17) with appropriate initial conditions represent a mathematical model of the measuring system. This constitutes the basis for testing our model. Thus the model together with relations (6), (7), (8) or (6), (10), (11) enables us to simulate measurements of the flow velocity and fluid temperature.

## 4. Methodology and simulation results

The purpose of testing our model is to analyze the process dynamics of switching between heating levels of the two-state hot-wire anemometer while minimizing the measurement cycle time. The model testing was implemented for the measuring system in which the controller gain is a function of flow velocity and of the given heating coefficient. For purposes of comparison, the classical system with the fixed-parameter controller was also subjected to simulated testing. The testing was carried out utilizing an iterative numerical solver on the system of equations that models the hot-wire anemometer. The MATLAB environment was utilized, and the system of differential equations was solved using the Runge-Kutta numerical method of the fifth order. The simulation process involved determining the steady-state condition for a given set of flow parameters and measuring system. It also involved calculating the system response following the sensor heating being switched to a higher level and then back to a lower level again. After each switching operation, we measured the time taken to re-establish a steady-state current for a given heating level. The flow velocity was also determined at this point.

Testing was performed using a simulated measuring sensor. The simulation included tungsten wire three micrometers in diameter within the modeled sensor apparatus. Air at a temperature of T_G_ = 293 K was input as the fluid medium for all testing. Parameters of the sensor and the measuring system used in the simulation were consistent with those used often found in hardware prototype measuring systems. Relevant parameters are listed in Tables 1 and 2.

**Figure 2.**
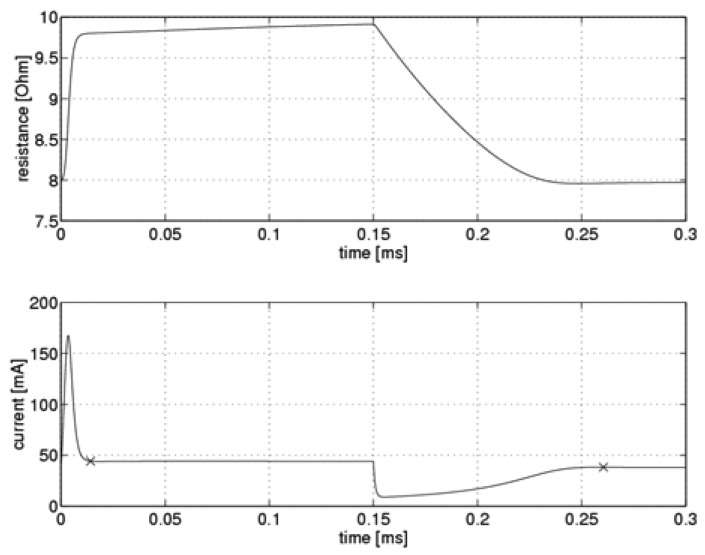
System simulation results.

During the first step of the simulation, the optimal values of the system controller gain k_C_ were determined for various flow velocities. “Optimal” refers to values for which a steady-state current could be established within the shortest possible time and with a fluctuation of less than 0.2 mA. The gain k_CH_ associated with switching to a higher heating level and the gain k_CL_ for switching to a lower one were each determined separately. Figure 2 presents simulation results from the system.

The figure illustrates the time-dependent variability of the sensor resistance and sensor current during the measurement cycle. Points at which the steady-state current was established are marked with an ”x”. The response shown in Figure 2 was obtained with a flow velocity of 10 m/s and using adjusted optimal values for the controller gain parameters. It is clear that as the sensor's temperature increased, the steady-state current was reached at lower resistance values. The sensor was heated up by a short pulse of high-intensity current, following which the current reached steady state. On the other hand, as the sensor cooled down, a rapid decrease in current intensity took place, following which the current slowly approached its steady state value asymptotically.

**Figure 3.**
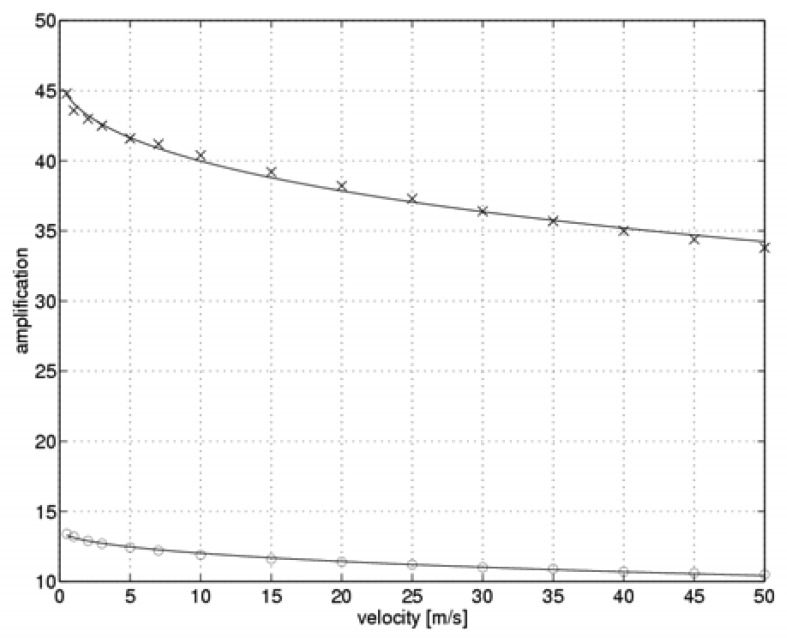
Simulated optimal values of the measuring system controller gains k_C_ (“x” - k_CH_, “o” - k_CL_)

Figure 3 shows optimal values for the measuring system controller gains k_C_ in the flow velocity function. The optimal values of the gain k_CH_ are marked with an “x”, while the optimal values of k_CL_ gain are marked with an “o”.

The least squares method was employed to fit the values obtained from the simulation to an analytical function of the form:

(18) $$k_{C}=\frac{k_{C0}}{\sqrt{1+\frac{V}{V_{C0}}}}.$$

The form of this function was chosen experimentally such that it would be possible to obtain a good fit to the data provided that a simple form of the function was supplied together with a minimal number of parameters. The values of the parameters in function (18) are k_C0_=13.71, V_C0_=500.3 m/s for the gain k_CH_, and k_C0_=46.28, V_C0_=403.8 m/s for the gain k_CL_. The fitted functions are shown in Figure 3 (solid line). Function (18) may be successfully implemented in the measurement algorithm of the real two-state anemometer in order to minimize the time required for a single measurement cycle.

**Figure 4.**
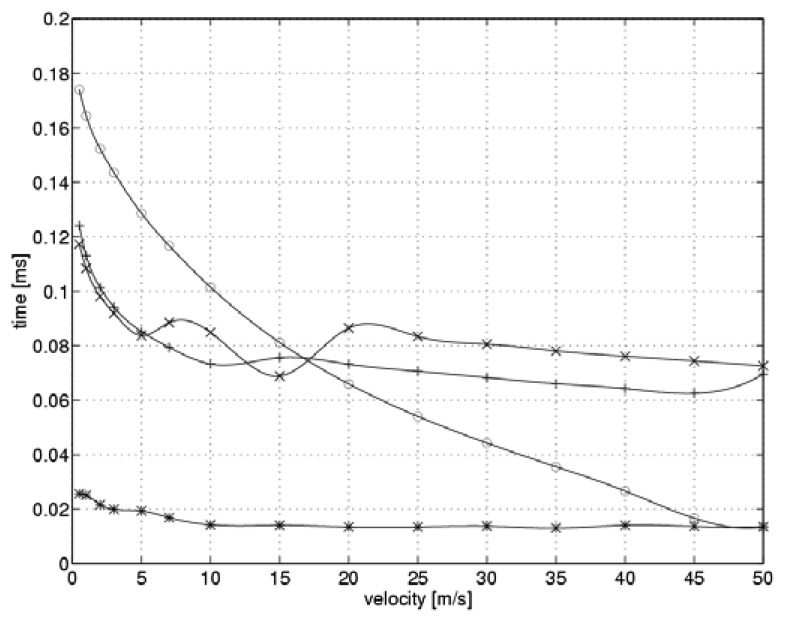
Reaching steady state after switching over to a higher heating level (“*” -optimal, “o” - minimum, “+” - intermediate, “x” – maximum gain value).

Figure 4 shows the simulated time required to establish a steady-state current as a function of flow velocity when the system switches over to a higher heating level.

The optimal values for controller gain shown in Figure 3 are marked by “*” symbols. For the purpose of comparison, we also present the situation when the controller gain remains constant during the measurement cycle. Three values of the gain were taken into account: the minimum and maximum values from a range of optimal values, and the intermediate value, which was optimal for the flow velocity of 10 m/s. “o” symbols indicate the minimum value of k_C_=10.5, while the “+” symbols highlight the intermediate value of k_C_=39.5. The “x” symbols indicate the maximum value of k_C_=44.8.

Analysis of the function shown in Figure 4 reveals that the relation between controller gain and the flow velocity allows for a significant reduction in the time needed to reach steady-state current levels during the sensor heating process.

**Figure 5.**
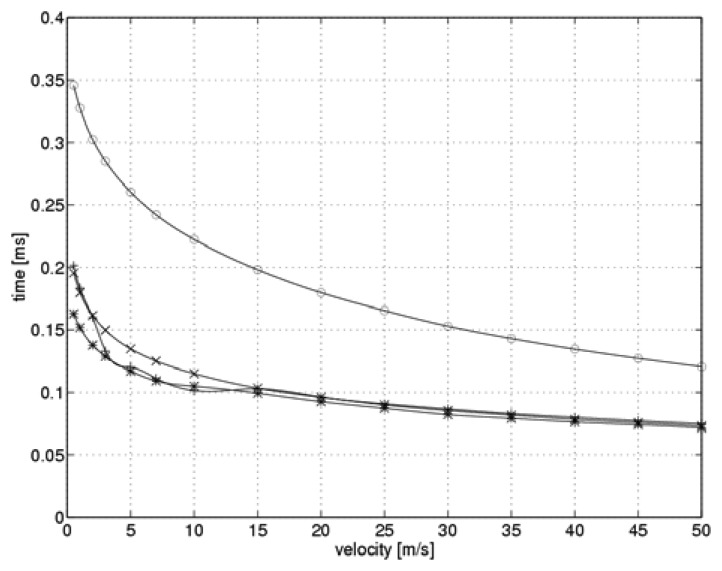
Reaching steady state when switching over to the lower heating level (“*” -optimal, “o” - minimum, “+” – intermediate, “x” – maximum gain value).

The reduced heating time was just 20% of that needed when a fixed gain controller was used. When the controller gain was set to its minimal value, the heating time was strongly related to the flow velocity. On the other hand, the relationships between heating time and flow velocity for intermediate and maximum values of controller gain were similar. Fluctuations over time in the case of the maximum controller gain can be explained by the non-linear nature of the measuring system simulation. The time required to establish a steady-state current as a function of flow velocity when switching over to the lower heating level is shown in Figure 5.

Similar to previous graphs, the optimal values of the controller gain are marked by “*” symbols. Points marked “o” denote a gain of k_C_=10.5, while the symbols “+” and “x” denote controller gains of k_C_=39.5 and k_C_=44.8, respectively. As shown in Figure 5, during sensor cooling the relation between the controller gain and the flow velocity allows for only an insignificant reduction in time required to reach steady state. At low velocities, this reduced time interval was about 80% of the time required to reach steady state when a fixed gain controller was used. On the other hand, the relationships for maximum and intermediate gains are similar to that obtained for the case of optimum controller gain. For the minimal controller gain, cooling time is strongly related to the flow velocity and is substantially longer than that of the other cases.

**Figure 6.**
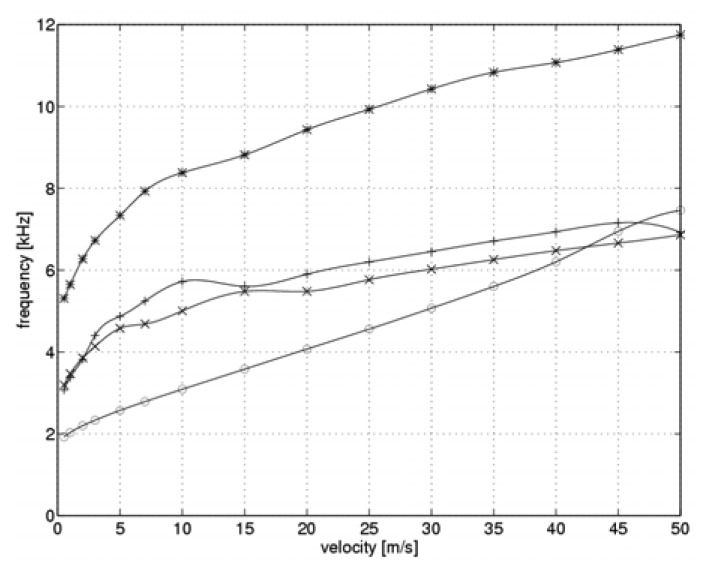
Frequencies during a full measurement cycle (“*” - optimal, “o” - minimum, “+” – intermediate, “x” – maximum gain value).

Based on the sensor heating and cooling times, our simulator could calculate frequencies for the full measurement cycle. Figure 6 shows the results in terms of the flow velocity.

Similar to previous graphs, the optimal values of the controller gain are marked by “*” symbols. Points marked “o” denote a gain of k_C_=10.5, while the symbols “+” and “x” denote controller gains of k_C_=39.5 and k_C_=44.8, respectively. Figure 6 shows that the relationship between the controller gain and the flow velocity allows for approximately a two-fold increase in measurement cycle frequency as compared to the frequency with a constant gain controller. This is also accompanied by a two-fold broadening of the measuring system transmission bandwidth and by a two-fold reduction of the measurement time. Measured value calculations were performed twice in each measurement cycle, once after each heating switch-over even. Accordingly, the actual frequencies for determining the measured values are double the frequencies shown in Figure 6. Moreover, Figure 6 reveals that given a fixed parameter gain, it is more beneficial to increase the controller gain. Under a given set of measurement conditions, the gain should always be adjusted in order to minimize the time taken for each measurement cycle.

**Figure 7.**
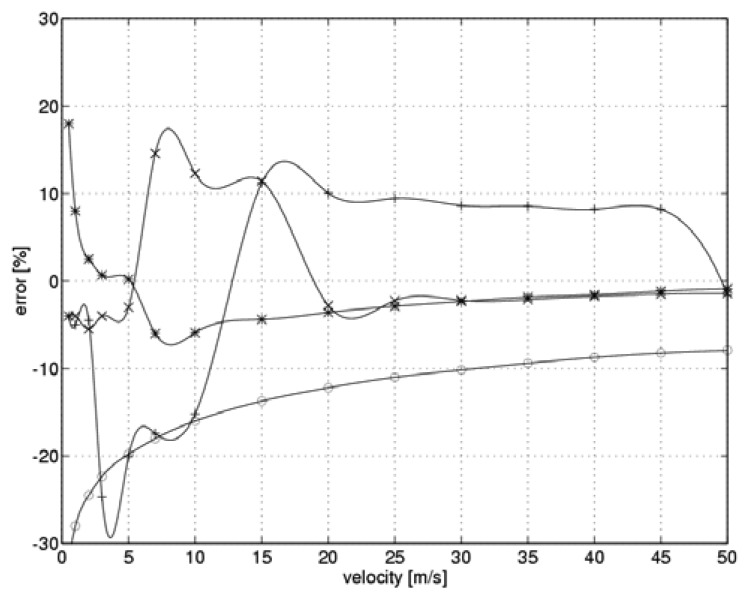
Relative error in velocity measurement during model testing (“*” - optimal, “o” -minimum, “+” – intermediate, “x” – maximum gain value).

In each of the aforementioned simulations, the measured flow velocity was determined consistent with the two-state hot-wire anemometer algorithm. Subsequently, the relative measuring errors were evaluated:

(19) $$\delta =\frac{v-V}{V}\times 100\%,$$

where: *V* – the preset velocity of the fluid, *v* – the determined velocity of the fluid. The results are shown in Figure 7. The “*” symbols refer to scenarios that involved the optimal value of the controller gain, while the symbols “o”, “ +” and “x” denote gain values of k_C_=10.5, 39.5 and 44.8, respectively. To allow for inter-experiment comparisons, the means of the absolute values of the relative errors and the respective standard deviations were also calculated. The results are shown in Table 3.

In our simulation, the measuring error during steady-state current measurement was the main source of our final flow velocity error. Consistent with the non-linear nature of the measuring system, these errors show a broad distribution. Data presented in Table 3 implies that the best results were obtained for the controller with a gain that had been adjusted consistent with the flow velocity and the sensor heating level. Other cases were characterized by significantly higher values of the mean relative error and the standard deviation. This can be explained in terms of the time derivatives of the sensor current as the system moves towards steady state.

## 5. Conclusions

In this study, we proposed a method for optimizing the transmission bandwidth of two-state hotwire anemometers. Our method is based on a specialized constant-temperature circuit with variable dynamic parameters and an appropriate measurement cycle strategy. Model testing was used to evaluate our method. Our results show a questionably significant broadening of the two-state hot-wire anemometer transmission bandwidth. In a case analyzed in this study, the dependence of the controller's gain on flow velocity and sensor heating levels limits the maximum measurement cycle frequency to 5 to 12 kHz. This range is twice the frequency obtained in the case of a model controller with fixed parameters. It is an order of magnitude faster than the maximum frequencies used to date in real-world measuring systems [17-19]. We believe that we can further improve our results by optimizing the measuring system and the measuring cycle strategy. Our system's improvement in transmission bandwidth is proportional to the increase in measuring cycle frequency.

The dependence of the controller's gain on flow velocity allows, for the optimization of sensor heating processes. As the sensor heats up, it is necessary to ensure that the rest of the electronic circuitry can acquire a sensor current commensurate with the heating effect. Given a fixed gain controller, a small gain value that is optimized for sensor heating at high flow velocities results in a significantly prolonged sensor cooling process.

The solution proposed in our study also allows for the minimization of measuring errors. The assumption underlying our method is that the flow velocity and temperature do not change significantly between successive switching events over a single measurement cycle. Increasing the switch-over frequency improves the validity of this assumption. In cases when the controller gain depends on flow velocity and sensor heating level, the measuring error is minimal during recording of the steady-state sensor current.

Implementing our proposed method for optimizing two-state hot wire anemometer transmission bandwidth requires that a very specific controlled constant-temperature circuit be integrated with the measurement algorithm. In the future, we plan to develop hardware and software that will allow us to construct a real-world model of our proposed solution. We also plan to experimentally validate theoretical results obtained from simulations.

## Figures and Tables

**Table 1.** Parameters of the simulated measuring sensor.

**R_0_**	**T_0_**	**α_0_**	**I_L_**	**V_L_**	**τ_L_**	**n**

[Ω]	[K]	[1/K]	[A]	[m/s]	[s]	
5	293	3.33×10^-3^	42×10^-3^	7.7	0.28×10^-3^	0.5

**Table 2.** Parameters of the simulated measuring system.

**R_I_**	**k_I_**	**η_1_**	**η_2_**	**U_0_**	**R_A_**	**k_A_**	***τ****_A_*	**R_C_**	***τ****_C_*

[Ω]				[V]	[Ω]		[s]	[Ω]	[s]
10	1	1.6	2.0	1×10^-6^	1×10^6^	1×10^6^	0.1	100	150×10^-6^

**Table 3.** Distribution parameters including relative error.

	**controller gain**			

	**optimal variable**	**minimal constant**	**intermediate constant**	**maximal constant**

**mean relative error [%]**	2.90	12.55	10.11	4.56
**standard deviation [%]**	2.65	4.69	11.02	6.18
